# Mutation in the Prothrombin Gene G20210A as a Cause of Cerebral Venous Thrombosis

**DOI:** 10.1155/2012/828050

**Published:** 2012-05-08

**Authors:** Jorge A. Arroyave, Jairo Quiñones

**Affiliations:** ^1^Internal Medicine, Universidad CES, Medellín, Colombia; ^2^Neuroinmunology, Clinical Neurology, Fundación Clínica Valle del Lili, Cali, Colombia

## Abstract

*Introduction*. Cerebral venous sinus thrombosis (CVST) is a rare form of cerebrovascular disease, which may manifest clinically by a wide variety of signs and symptoms. It has been associated with multiple risk factors including genetic or acquired blood disorders, infections, and trauma. *Case Report*. Man of 17 years who presented with 10 days of intense global headache with nausea and vomiting and subsequent onset of mild hemiparesis and hypoesthesia in right hemibody. Studies show venous thrombosis of the superior longitudinal sinus. It was identified a gene mutation in prothrombin G20210A as a probable cause of the thrombosis. *Conclusions*. Substitution of guanine for adenine at nucleotide 20210 in the coding region of the prothrombin gene is the second most common primary thrombophilia. Multiple cases of CVST have been associated with this mutation. In the presence of CVST must be considered the primary studies for thrombophilia gene mutations, including prothrombin G20210A.

## 1. Introduction

The cerebral venous sinus thrombosis (CVST) is a rare form of stroke corresponding to about 0.5% to 1% of all cases presenting with this condition. Annual incidence is estimated at about 3 to 4 cases per 1 million people, being more common in women, particularly between the ages of 25 and 35 [1–4].

In the last decades has been increased the number of diagnoses of this condition probably due to the improvement in neuroimaging studies, allowing an appropriate treatment with good outcomes in more than 80% of patients [3].

CVST can clinically manifest with a wide variety of symptoms ranging from mild headache to severe neurological alterations. Two main mechanisms that explain the signs and symptoms include cerebral venous thrombosis with local effects of obstruction and venous sinus thrombosis leading to intracranial hypertension [3].

The diagnosis should be suspected in young patients with recent headache or unusual symptoms similar to those of cerebrovascular disease by arterial thrombosis. In general, the average time to reach this diagnosis is 7 days according to published reports [5]. The most sensitive test for the diagnosis is the cerebral magnetic resonance with venography [2].

Multiple risk factors have been associated with CVST but few are reversible. In 85% of cases can be identified a risk factor or a direct cause of thrombosis as genetic or acquired prothrombotic alterations, infectious diseases, inflammatory diseases, hematological conditions, medications, and trauma [2, 3].

Important genetic prothrombotic conditions include deficiency of antithrombin, protein C or S; factor V Leiden, prothrombin G20210A mutation, and hyperhomocysteinemia. The prothrombin G20210A mutation has been associated with venous thrombotic events in unusual places including cerebral venous sinus. This occurs mainly in women using oral contraceptives or in physiological states like pregnancy. We describe an unusual case of CVST in a young man who has the prothrombin G20210A mutation.

## 2. Case Report

**Figure 1:**
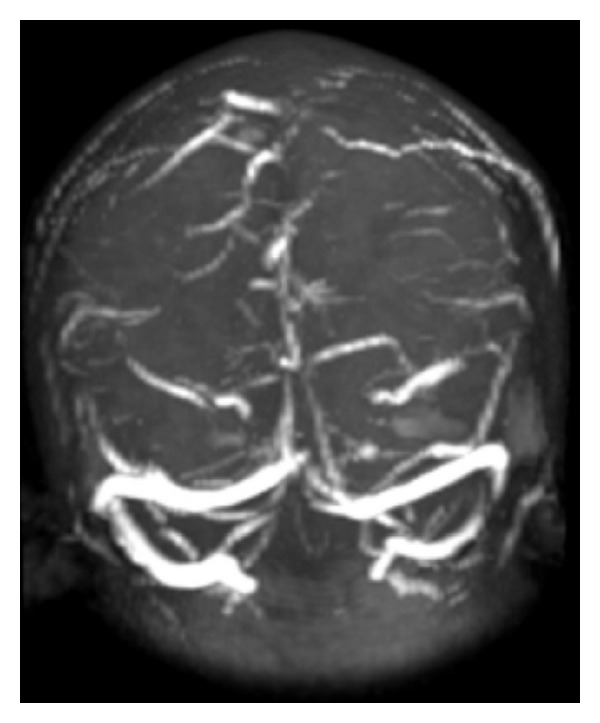
Magnetic resonance venography of the brain with superior longitudinal sinus thrombosis.

**Figure 2:**
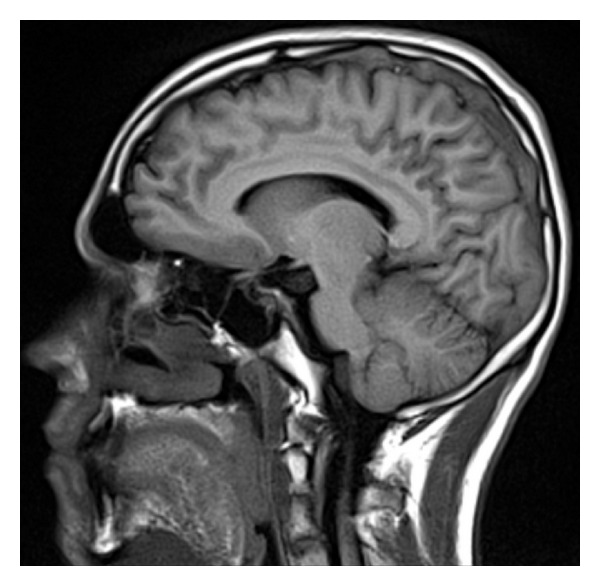
Sagittal T1 with superior longitudinal sinus thrombosis.

A 17-year-old man, high school student, with no relevant medical history was admitted for 10 days of intense global headache accompanied by nausea and vomiting. He was hospitalized for 4 days before admission at another institution, where imaging studies were performed with simple brain scan reported as normal and then discarded central nervous system infection. The patient was managed with analgesics and was discharged. For persistence of the headache and sensory symptoms including hypoesthesia in right hemibody with slight loss of muscle strength, he was admitted again. He was evaluated by the Neurology Department with a physical examination which showed a blood pressure of 138/76, heart rate of 88 beats per minute, and respiratory rate of 17 per minute, temperature of 37 degrees, neurological exam with fluent speech, cranial nerves normal, fundus with absence of venous pulses bilaterally, muscle strength of 3 of 5 in right hemibody, tendon reflexes of +++ numbness in right hemibody. No nuchal rigidity or other signs of meningitis were identified. A new magnetic resonance imaging study with gadolinium and venography reported cerebral sinus thrombosis of the superior longitudinal sinus with venous infarction in subacute phase (Figures 1 and 2). Given the findings, it was decided to initiate anticoagulation with unfractionated heparin and warfarin. Further investigations ruled out infectious etiology. He was studied for primary thrombophilia identifying a mutation in the prothrombin gene with the following test: “Gen F2 was analyzed using quantitative chain reaction (qPCR) with amplimers and subsequent extension of thermal dissociation curve. Platform Roche Light Cycler. It confirms the presence of G20210A mutation in the prothrombin gene. The mutation was found in heterozygous state, means that the patient has copy of the mutated gene.”

The patient has a total resolution of symptoms and continues with oral anticoagulation with warfarin.

## 3. Discussion

The prothrombin gene is located on chromosome 11p. Substitution of guanine for adenine at nucleotide 20210 in the coding region of this gene generates increased levels of prothrombin, the precursor of thrombin, which is essential component of homeostasis. It is suggested that the increase in prothrombin levels is the cause of the hypercoagulable state, generating increased risk of venous thrombotic events [6]. The prevalence of the mutation is between 0.5% and 4%, mainly present in heterozygous state. Homozygous presentation is very rare, occurring in 1 in 4000 Caucasian individuals.

In 1996, Poort et al. described for the first time the association of the mutation in the prothrombin gene with increased plasma levels of this, with the resulting increase in venous thrombosis [7]. In 1998, it was reported the first case of CVST associated with the prothrombin G20210A mutation by Bloem et al. [8]. From this date, there have been multiple reports in patients with different clinical features, usually with additional triggers such as pregnancy, oral contraceptives, among others. In 2006, it was performed a meta-analysis that included 360 patients with CVST, finding that the OR for this mutation reached 9.27 (95% CI 5.85 to 14.67), which is an important association with venous thromboembolism in general [9].

This paper highlights the case of a young patient with an intense global headache, in which at 10 days after onset, CVST was demonstrated. The time to the diagnosis, although prolonged, is in the average range as published in the literature. This case has unusual aspects, mainly because of being male patient without precipitating factors in whom evidenced as the only possible etiology of thrombosis the presence of the mutation in the gene prothrombin G20210A. The patient responded appropriately to anticoagulant therapy with complete resolution of symptoms.

## 4. Conclusions

It is important to consider the diagnosis of CVST in young patients with recent onset of severe headache and make appropriate studies in search of their cause including primary thrombophilia testing. The mutation of prothrombin gene appears as a factor that should always be considered for proper diagnosis and treatment.
